# The Lung Microbiome of Three Young Brazilian Patients With Cystic Fibrosis Colonized by Fungi

**DOI:** 10.3389/fcimb.2020.598938

**Published:** 2020-11-11

**Authors:** Otávio Guilherme Gonçalves de Almeida, Carolina Paulino da Costa Capizzani, Ludmilla Tonani, Patrícia Helena Grizante Barião, Anderson Ferreira da Cunha, Elaine Cristina Pereira De Martinis, Lidia Alice Gomes Monteiro Marin Torres, Marcia Regina von Zeska Kress

**Affiliations:** ^1^ Departamento de Análises Clínicas, Toxicológicas e Bromatológicas, Faculdade de Ciências Farmacêuticas de Ribeirão Preto, Universidade de São Paulo, Ribeirão Preto, Brazil; ^2^ Departamento de Genética e Evolução, Centro de Ciências Biológicas e da Saúde, Universidade Federal de São Carlos, São Carlos, Brazil; ^3^ Hospital das Clínicas da Faculdade de Medicina de Ribeirão Preto, Universidade de São Paulo, Ribeirão Preto, Brazil

**Keywords:** cystic fibrosis, lung microbiome, metagenomic, colonization, *Aspergillus fumigatus*, *Burkholderia*, lung microbiota

## Abstract

Microbial communities infiltrate the respiratory tract of cystic fibrosis patients, where chronic colonization and infection lead to clinical decline. This report aims to provide an overview of the diversity of bacterial and fungal species from the airway secretion of three young CF patients with severe pulmonary disease. The bacterial and fungal microbiomes were investigated by culture isolation, metataxonomics, and metagenomics shotgun. Virulence factors and antibiotic resistance genes were also explored. *A. fumigatus* was isolated from cultures and identified in high incidence from patient sputum samples. *Candida albicans, Penicillium* sp., *Hanseniaspora* sp., *Torulaspora delbrueckii*, and *Talaromyces amestolkiae* were isolated sporadically. Metataxonomics and metagenomics detected fungal reads (*Saccharomyces cerevisiae*, *A. fumigatus*, and *Schizophyllum* sp.) in one sputum sample. The main pathogenic bacteria identified were *Staphylococcus aureus*, *Pseudomonas aeruginosa*, *Burkholderia cepacia* complex, and *Achromobacter xylosoxidans*. The canonical core CF microbiome is composed of species from the genera *Streptococcus, Neisseria, Rothia, Prevotella*, and *Haemophilus*. Thus, the airways of the three young CF patients presented dominant bacterial genera and interindividual variability in microbial community composition and diversity. Additionally, a wide diversity of virulence factors and antibiotic resistance genes were identified in the CF lung microbiomes, which may be linked to the clinical condition of the CF patients. Understanding the microbial community is crucial to improve therapy because it may have the opposite effect, restructuring the pathogenic microbiota. Future studies focusing on the influence of fungi on bacterial diversity and microbial interactions in CF microbiomes will be welcome to fulfill this huge gap of fungal influence on CF physiopathology.

## Introduction

Cystic fibrosis (CF) is the most common lethal autosomal recessive disorder in Caucasians and is characterized by a mutation in the cystic fibrosis transmembrane conductance regulator gene (CFTR) (Cutting, 2015; Lavelle et al., 2016). The codified CFTR protein is responsible for the transport of chloride ions over the apical membranes of epithelial cells from tissues such as airways, intestine, pancreas, kidneys, sweat glands, and male reproductive tract (Bell et al., 2020). In the sinopulmonary tract, the transport of chloride anions contributes to the hydration of the mucus, forming a physical barrier against microbial pathogens and particles, which can be expelled by expectoration or swallowed through the action of ciliary movements in the trachea (Patel et al., 2020). In addition, the CFTR protein also transports bicarbonate ions, controls the levels of acidity in fluid secretions, and interacts with other membrane proteins to maintain tight junctions. All these roles of CFTR protein allow mucociliary clearance (MCC), whose impairment leads to the production of more viscous mucus, predisposing patients to develop chronic infections of airways (Procianoy et al., 2020). As a result, acute episodes of lung infections are observed in CF, receiving the general designation of “pulmonary exacerbation” (De Koff et al., 2016).

To overcome the deficiency in MCC and to prevent microbial colonization in the respiratory tract, the prescription of inhaled and systemic antibiotics is the first line of treatment available (De Koff et al., 2016). Pulmonary microbial colonization is facilitated by the building up of mucus, which may be a nutrient source for anaerobic bacteria such as *Streptococcus, Prevotella*, and *Veilonella*, whose metabolism releases short-chain fatty acids that serve as substrates for *Pseudomonas aeruginosa*. These bacteria are recognized as keystone taxa because they facilitate the growth of canonical CF pathogens through cross-feeding pathways (Silveira et al., 2020; Flynn et al., 2020). However, despite the frequent detection of these taxa in CF patients, a very high inter-patient variability of the airway microbiome has been reported (Bacci et al., 2020).

The CF microbiome varies according to age and clinical conditions of each patient, and it is generally more diverse in the younger than in the elderly, with decreasing diversity directly correlated to the loss of pulmonary function (Cox et al., 2010; Coburn et al., 2015; Caverly and Lipuma, 2018). Studies have also pointed out fungi of the genera *Candida* and *Aspergillus* as members of CF microbiomes, mainly related to cases of pulmonary exacerbation (Bevivino et al., 2019; Soret et al., 2020). However, research and treatment have conventionally focused on bacterial pathogens with little attention to fungal species. The fungi have been sub-notified as agents of CF infections for several reasons: (i) they might be erroneously regarded as contaminants in laboratory cultures; (ii) there are usually limited resources to recover fungal species in routine analysis of clinical samples; (iii) there is a lack of robust clinical guidelines and laboratory tools to assure unambiguous taxonomic identification of fungi; and (iv) clinicians tend to get puzzled by laboratory reports on unfamiliar fungal genera, which leads to difficulties in evaluating their real significance concerning the clinical findings in CF cases (Martín-Gómez, 2020)⁠.

In the era of next-generation sequencing (NGS) technologies, knowledge about the microorganisms related to CF has greatly enhanced. Previously overlooked microorganisms have been revisited, and new paradigms emerged in the CF microbiome. Knowledge of microbial composition has been correlated with the degree of lung function and contributes to a more assertive clinical diagnosis (Coburn et al., 2015; Caverly and Lipuma, 2018; Quinn et al., 2019).

Taking into consideration the increased detection of fungal infections in patients with CF and the novel tools available to evaluate the microbiome, this study was carried out to describe the lung microbial diversity of three young CF patients who were culture-positive for filamentous fungi at different stages of the disease.

## Casuistic and Methods

### Participants and Sample Collection

Three young patients with CF were recruited at the ‘Ambulatório Multidisciplinar de Fibrose Cística (AMFC)’ at the ‘Hospital das Clínicas da Faculdade de Medicina de Ribeirão Preto da Universidade de São Paulo (HC-FMRP-USP)’ during 2018. The patients were unequivocally diagnosed with CF, either by detection of two mutations in the CFTR gene or by an abnormally high result for the sweat chloride test (≥60 mmol/L). They were enrolled due to their high visit frequency to the hospital, severe lung disease and presented a history of fungal isolation in sputum and chronic infections by pathogenic bacteria. They were named patient A (male, 12 years old), patient B (female, 9 years old), and patient C (male, 17 years old). The patients also presented the loss of pulmonary function, according to the spirometric parameters (% of predicted forced expiratory volume in one second, FEV1%), with test results for patients A, B, and C of 39, 64, and 37%, respectively. During the study period, the patients received antibiotics for pulmonary exacerbation (**Supplementary Table S1**). The patients were followed up to seven months, with a collection of sputum samples during routine clinical appointments and/or during hospitalization due to pulmonary exacerbation. A total of twelve sputum samples were collected with an average interval of 44 days and were distributed as follows: five from patient A (PA1 to 5), three from patient B (PB1 to 3), and four from patient C (PC1 to 4). The sputum samples were collected by expectoration into a sterile cup after mouth rinsing with water to prevent excessive salivary contamination. To each sputum sample, an equal volume of sterile dithiothreitol (DTT) solution (DTT 50 µg ml^−1^ dissolved in phosphate-buffered saline plus 0.1% gelatin) was added. After 30 min, the samples were vortexed to homogenize and cultured for bacterial isolation by seeding on blood agar, chocolate agar, salt mannitol agar, MacConkey agar, and *Burkholderia cepacia* selective agar at the routine clinical laboratory of the HC-FMRP-USP, with incubation at 37°C, under anaerobiosis for 24 to 48 h. Fungal isolation was performed by plating on Sabouraud Dextrose Agar (SDA—Acumedia, Michigan, USA) with 50 µg ml^−1^ chloramphenicol and in SDA with 50 µg ml^−1^ chloramphenicol plus 500 µg ml^−1^ cycloheximide. Seeded plates were incubated at both 25 and 37°C for up to three weeks. The remaining sputum samples were frozen at −80°C until DNA extraction for metagenomics analysis

### Ethics Statement

The sputum samples from the three patients who volunteered for the study were collected at HC-FMRP-USP following the ethical guidelines of the hospital. This study was approved by the Ethics Committee of “Faculdade de Ciências Farmacêuticas de Ribeirão Preto da Universidade de São Paulo” (FCFRP-USP), under protocol number 2.492.043, in accordance with the HC-FMRP-USP as a co-participating institution. The parents of the patients signed a term of Written Informed Consent to permit the participation of the teenagers in this study.

### Bacterial Identification and Molecular Identification of Fungal Isolates

The bacterial isolates were biochemically identified by VITEK^®^2 (bioMérieux, France) at HC-FMRP-USP. Fungal colonies were identified by conventional methods and DNA sequencing of the internal transcribed spacer (ITS) region. *Aspergillus fumigatus* was additionally identified by the sequencing of calmodulin (cmd)- and beta-tubulin (*benA*)-encoding genes. Briefly, fungal DNA was extracted as previously described (Tonani et al., 2018) and used for PCR amplification with Phusion High Fidelity DNA Polymerase (New England BioLabs Inc.) with the primers ITS1 and ITS4 for ITS (White et al., 1990); *cmd5* and *cmd6* for calmodulin (Hong et al., 2005); and *bt2a* and *bt2b* for *β*-tubulin (Glass and Donaldson, 1995). PCR-purified products were sequenced with the same primers in the ABI3730 DNA Analyzer (Applied Biosystems). Each DNA sequence was analyzed with ChromasPro Software (ChromasPro1.7.6, Technelysium Pty. Ltd., Tewantin QLD, Australia) and tested against publicly available DNA sequences in the NIH genetic sequence database (Altschul et al., 1990).

### Metagenomic DNA Extraction

The total DNA from sputum samples was extracted using the commercial Quick-DNA Fungal/Bacterial Miniprep kit (Zymo Research, USA) according to the instructions of the manufacturer. DNA quality and quantity were measured by a Qubit^®^ 3.0 fluorometer (Thermo Fisher Scientific, USA) following standard methods provided by the manufacturer.

### Preparation of Samples for Metataxonomic Analysis

To assemble the community taxonomic composition of bacteria and fungi, the 16S *rRNA* V3–V4 and ITS2 (Internal Transcribed Spacer) regions were amplified from aliquots of 1.0 μl from the whole genomic DNA sample extract.

The 16S *rRNA* V3–V4 regions were amplified by a first PCR employing the universal primers: forward 5′–TCGTCGGCAGCGTCAGATGTGTATAAGA GACAGCCTACGGGNGGCWGCAG–3′, and reverse GTCTCGTGGGCTCG GAGATGTGTGTGTATAAGAGACAGGACTACHVGGGTATCTAATCC–3′. The reaction media of each PCR contained 10 μl of GoTaq^®^ Colorless Master Mix 2× (Promega, USA), 1.0 μl of metagenomic DNA and 0.3 μM of forward and reverse primers. The final volume of 20.0 μl was achieved by completing the reactional volume with ultrapure water.

The PCR conditions were characterized by a first denaturation step at 94°C/3 min followed by 25 cycles of denaturation at 94°C/30 s, annealing at 55°C/30 s, extension at 72°C/30 s, and a final extension step at 72°C/5 min.

To evaluate the quality and to confirm the amplification, agarose gel 2% (w/v) electrophoresis stained with UniSafe Dye 0.003% (v/v) was performed. The average amplicon size obtained for each sample was 500 bp.

Regarding the ITS2 amplification run for fungi, the forward 86-F 5′-TCGTCGGCAGCGTCAGATGTGTATAAGAGACAGGTGAATCATCGAATCTTTGAA-3′ and reverse ITS4R 5′-GTCTCGTGGGCTCGGAGATGTGTATAAGAGACAGTC CTCCGCTTATTGATATGC-3′ were used. The PCR components were the same as described above for 16S *rRNA* V3–V4 region amplification, although the PCR conditions to amplify ITS regions were slightly different: initially, a denaturation cycle at 95°C for 5 min was performed, followed by 35 cycles of denaturation at 95°C for 30 s, annealing at 56°C for 40 s, extension at 72°C for 1 min, and a final extension step at 72°C for 5 min. All PCRs were conducted on a Veriti™ Thermal Cycler (Applied Biosystems, USA).

The integrity checking of the generated amplicons was performed by agarose gel electrophoresis 2% (m/v) stained with UniSafe Dye 0.003% (v/v). The average amplicon size obtained for each sample was 400 bp.

The PCR amplicons were subsequently purified using Agencourt AMPure XP magnetic beads (Beckman Coulter, USA) following the manufacturers’ guidelines.

The indexation step, required to pool the samples and to insert them into the flow cell, was performed through a second PCR for the entire set of generated amplicons. In this reaction, the adapter’s sequences were inserted in the amplicon extremities. PCR was conducted with the Nextera XT index kit (Illumina^®^) following the manufacturers’ instructions.

The resulting libraries were purified using magnetic beads provided in the Agencourt AMPure XP kit (Beckman Coulter) to remove fragments <100 bp and residual primers. The libraries were quantified by RT-PCR using the KAPA-KK4824 Library Quantification Kit (Illumina^®^) according to the manufacturer’s recommendations. Next, an equimolar pool of samples was prepared by normalization of all samples to the final concentration of 3 nM. High-throughput DNA sequencing (HTS) was conducted on a MiSeq platform (Illumina^®^).

### Metagenomic Sample Preparation and DNA Sequencing

The library preparation for metagenomics analysis was performed using the Nextera DNA Library Prep kit (Illumina^®^) to randomly fragment the metagenomic DNA using transposase activity. The adapters were inserted using the Nextera XT Index kit (Illumina^®^) following the manufacturers’ instructions. The libraries were quality-assured by removal of residual adapters, exceeding dNTPs and fragments <100 bp using AMPure XP magnetic beads (Beckman Coulter).

The libraries were quantified by RT-PCR using the KAPA-KK4824 Library Quantification Kit (Illumina^®^) following fabricant standards. A final concentration of 4 nM was used to normalize the libraries to pool them into the sequencer flow cell. The HTS was conducted on a NextSeq 500 platform (Illumina^®^) using the running kit NextSeq 500 MID Output (300 cycles) to generate 2 × 150 bp paired-end (PE) reads.

### Raw Reads Processing: Contaminant Removal and Quality Control

The first preprocessing step was to overlap the PE reads using the BBmerge tool (Bushnell et al., 2017)⁠. The FASTQ files derived from the metataxonomic approach were addressed by the BaseSpace (Illumina^®^) pipeline with reads without adapters and Phred score values higher than 30, so these files were not subjected to any further quality processing.

On the other hand, the FASTQ files derived from metagenomic DNA sequencing were quality trimmed using the BBDUK tool to remove adapters and fragments <100 bp and Phred score <30 (parameters: hdist = 1, tpe, tbo, qtrim = rl, trimq = 30, maq = 30, minlen = 100).

Due to intrinsic characteristics, the sputum matrix was heavily concentrated with DNA from human cells. Thus, the human DNA reads were removed for the final processing of the Metagenomics Shotgun data. This was performed by downloading the human reference genome (version GRCh38.p12) from the NCBI database and indexing it on the Bowtie2 tool (Langmead and Salzberg, 2012). The FASTQ files were aligned against the indexed reference genome, resulting in two separate files: one with matches and the other with no matches. The unmatched FASTQ files were selected for downstream metagenomics analysis.

### Community Taxonomic Composition

The community taxonomical composition was determined by two independent methods: (i) the QIIME1 open-reference method for amplicon analysis and (ii) MetaPhlan2-specific marker gene analysis for the metagenomics-derived data.

In the QIIME1 environment version 1.9.1 (Caporaso et al., 2010), the FASTQ files derived from metataxonomic HTS were converted to FASTA files, and the open-reference method was chosen to perform OTU (operational taxonomic units) picking using the UCLUST algorithm (Edgar, 2010) for sequence clustering. For fungal OTU picking, the UNITE database (version 8.0) was selected as a reference (Nilsson et al., 2019), and for bacterial OTU picking, the last version of the SILVA database (version 132) preformatted for QIIME was chosen (Quast et al., 2013).

The OTU tables were filtered to remove OTUs lower than 0.1% in relative abundance using built-in scripts provided by the pipeline. A bar plot of bacterial composition per patient was drawn in the R environment using the ggplot2 package. The statistical analyses related to alpha and beta-diversity measurements were performed on Microbiome Analyst (Dhariwal et al., 2017) environment selecting the default parameters of the “Marker Data Profiling (MDP)” pipeline.

The MetaPhlan2 pipeline (Segata et al., 2012) was chosen to process the shotgun-derived data using default parameters.

### Annotation of Virulence and Antibiotic Resistance Genes

The search for virulence factors (VFs) in the genomes was conducted using the ShortBRED (Short, Better Representative Extract Dataset) tool (Huttenhower et al., 2012). Initially, a FASTA file containing the entire set of VFs curated on the Victors Database (Sayers et al., 2019)⁠ was downloaded. Then, the UniRef90 database (Suzek et al., 2007) was downloaded and used as a comprehensive reference database for additional comparisons of protein sequences. To create a VF custom reference database, first, the script ShortBRED-Identify was used. This script performs a global sequence homology identification of protein families related to the target sequences from the Victors Database. These sequences were collapsed to form a unique consensus sequence that possesses short peptide markers that best represented a given protein family. Moreover, applying the script ShortBRED-Quantify, the reads were aligned against the custom reference database represented by the newer protein markers, determined by the ShortBRED-Identify algorithm, quantified in terms of their absolute counts, normalized by marker gene length and finally represented by reads per kilobase per million (RPKM).

Regarding the search of antibiotic resistance genes (ARGs) in the metagenome, the same methodology for VF determination was employed, differing only due to the reference database chosen, which was the CARD (Comprehensive Antibiotic Resistance Database) (Jia et al., 2017).

## Results

Three young patients with cystic fibrosis who were part of a long-term follow-up in the “Ambulatório Multidisciplinar de Fibrose Cística (AMFC)” at HC-FMRP-USP were monitored in this study in 2018. The youngest patient (patient B) had a fatal outcome due to severe and difficult-to-treat infection at the beginning of 2019.

The bacterial and fungal microbiotas/microbiomes of the patient’s lung were investigated by culture isolation, metataxonomics, and metagenomics shotgun sequencing. The culturable bacteria detected in the specimens of CF patients were for the patient A—methicillin-resistant *Staphylococcus aureus* (MRSA), *Pseudomonas aeruginosa*, and *Burkholderia cepacia* complex; for the patient B—*B. cepacia* complex; and for the patient C—*Staphylococcus aureus*, *P. aeruginosa, Stenotrophomonas maltophilia, Achromobacter* sp., *Achromobacter xylosoxidans*, and *Chryseobacterium indologenes* (**Supplementary Table S1**). The results of fungal culture revealed the frequent isolation of *Aspergillus fumigatus* from the sputum of patients A and C. Additionally, *Penicillium* sp. and *Torulaspora delbrueckii* were isolated from patient A, and *Candida albicans* was isolated from patient C. From patient B, *Penicillium* sp., *Hanseniaspora* sp., *Torulaspora delbrueckii*, and *Talaromyces amestolkiae* were isolated from sputum samples (**Table 1** and timeline **S1**). Regarding *A. fumigatus* clinical isolates, nucleotide polymorphisms were observed in calmodulin (cmd) and beta-tubulin (tub) genomic DNA sequences, which indicated four different sequence patterns, which we named sequence types (ST) 1 to ST4. Patient A had ST1 isolated from sputum samples PA1 and PA5 and ST2 from sputum samples PA3 and PA5. Patient B had no *A. fumigatus* isolates. Patient C had ST1, ST2, and ST3 isolated from sputum sample PC1, and ST4 from sputum samples PC1, PC2, PC3, and PC4 (**Table 2**). Focusing on sequence types, ST1 and ST2 were isolated from two different sputum samples from patient A, and ST4 was isolated from 4 sputum samples from patient B, thus indicating the colonization of patients A and C by *A. fumigatus* strains.

**Table 1:** Molecular identification of fungal isolates.

Patient	Sputum sample	ID	Species	ITS				Calmodulin				β-tubulin			
				Ident. (%)	e-Value	Max score	Access GenBank	Ident. (%)	e-Value	Max score	Access GenBank	Ident. (%)	e-Value	Max score	Access GenBank
PA	PA1	LMC8001.01	*Aspergillus fumigatus*	100	0.0	933	KC689325.1	100	0.0	952	MK451408.1	100	0.0	917	KJ175502.1
	PA2	NI	NI	–	–	–	–	–	–	–	–	–	–	–	–
	PA3	LMC8001.03	*A. fumigatus*	100	0.0	933	KC689325.1	100	0.0	952	MK451394.1	100	0.0	917	KJ175502.1
	PA4	LMC8001.08	*Penicillium* sp.	99.81	0.0	968	MK226539.1	–	–	–	–	–	–	–	–
	PA5	LMC8001.09	*A. fumigatus*	100	0.0	933	KC689325.1	100	0.0	952	MK451408.1	100	0.0	917	KJ175502.1
	PA5	LMC8001.10	*A. fumigatus*	100	0.0	933	KC689325.1	100	0.0	952	MK451394.1	100	0.0	917	KJ175502.1
	PA5	LMC8001.12	*Torulaspora delbrueckii*	100	0.0	1360	KY105646.1	–	–	–	–	–	–	–	–
PB	PB1	NI	NI	–	–	–	–	–	–	–	–	–	–	–	–
	PB2	LMC8002.01	*Penicillium* sp.	98.48	0.0	926	MH864712.1	–	–	–	–	–	–	–	–
	PB2	LMC8002.02	*Hanseniaspora* sp.	99.37	0.0	1,146	AJ512437.1	–	–	–	–	–	–	–	–
	PB2	LMC8002.03	*Torulaspora delbrueckii*	100	0.0	1,362	KY105646.1	–	–	–	–	–	–	–	–
	PB3	LMC8002.04	*Talaromyces amestolkiae*	100	0.0	961	MH856395.1	–	–	–	–	–	–	–	–
PC	PC1	LMC8003.01	*A. fumigatus*	100	0.0	963	KC689325.1	100	0.0	896	MK451402.1	100	0.0	917	KJ175502.1
	PC1	LMC8003.02	*A. fumigatus*	100	0.0	963	KC689325.1	99.79	0.0	952	MK451394.1	99.8	0.0	924	KJ175502.1
	PC1	LMC8003.06	*A. fumigatus*	100	0.0	963	KC689325.1	100	0.0	952	MK451394.1	100	0.0	917	KJ175502.1
	PC1	LMC8003.09	*Candida albicans*	100	0.0	924	KY101918.1	–	–	–	–	–	–	–	–
	PC1	LMC8003.10	*A. fumigatus*	100	0.0	963	KC689325.1	100	0.0	952	MK451408.1	100	0.0	917	KJ175502.1
	PC2	LMC8003.11	*A. fumigatus*	100	0.0	963	KC689325.1	99.79	0.0	952	MK451394.1	99.8	0.0	924	KJ175502.1
	PC3	LMC8003.13	*A. fumigatus*	100	0.0	963	KC689325.1	99.79	0.0	952	MK451394.1	99.8	0.0	924	KJ175502.1
	PC4	LMC8003.14	*A. fumigatus*	100	0.0	963	KC689325.1	99.79	0.0	952	MK451394.1	99.8	0.0	924	KJ175502.1

ID, fungal idetification; NI, no isolation; -, not done; Ident., identity; ITS, internal transcribed spacer.

**Table 2:** *A. fumigatus* clinical isolate nucleotide polymorphism.

Patient	Sample	ID	Gene (bp)				ST
			cmd	cmd	cmd	tub	
			195	360	568	328	
**PA**	PA1	LMC8001.01	T	T	A	G	1
	PA3	LMC8001.03	C	G	G	G	2
	PA5	LMC8001.09	T	T	A	G	1
	PA5	LMC8001.10	C	G	G	G	2
**PC**	PC1	LMC8003.01	T	G	A	G	3
	PC1	LMC8003.02	C	G	A	A	4
	PC1	LMC8003.06	C	G	G	G	2
	PC1	LMC8003.10	T	T	A	G	1
	PC2	LMC8003.11	C	G	A	A	4
	PC3	LMC8003.13	C	G	A	A	4
	PC4	LMC8003.14	C	G	A	A	4

ID, strain identification; cmd, calmodulin; tub, ß-tubulin; ST, sequence type.

Concerning the results of HTS, for metataxonomic characterization of sputum samples from CF patients A, B, and C, the BaseSpace pipeline (Illumina^®^) returned an average of 153,229 and 87,538 quality-processed PE reads of 16S *rRNA* and ITS, respectively. These reads were overlapped to be analyzed on the QIIME1 pipeline, which is useful for determining the microbial community composition and diversity. A low overlap was observed, and only R1 FASTQ files were maintained, following the guidelines of the BBmerge tool, which recommends working only with the R1 reads to assure high quality during downstream processing if there is <15% overlap.

Regarding the HTS of metagenomics shotgun data, the average number of raw PE reads generated was 10,601,533 per sample. All samples were overlapped and preprocessed to remove low-quality base calls and adapter sequences, resulting in an average of 4,540,817 quality-processed PE reads per sample. However, the majority of community DNA was derivate from the hosts (mean of 95.82%) remaining an average of 182,342 quality-filtered PE reads by sample for taxonomic and functional downstream analyses.

### Community Composition Shifts

The structures of the microbial communities from CF patients were drawn from metataxonomic and metagenomic results to exploit the best of each technique, aiming to improve the resolution of the study. The metataxonomics focus on specific taxonomic markers, and the targeted amplification of these DNA regions allows for enrichment of OTUs that might otherwise be difficult to detect due to low relative abundances in shotgun datasets (Almeida and De Martinis, 2019). On the other hand, the analyses of single-copy species-specific reads from the shotgun results help in the assignment of OTUs at the species level.

**Figure 1:**
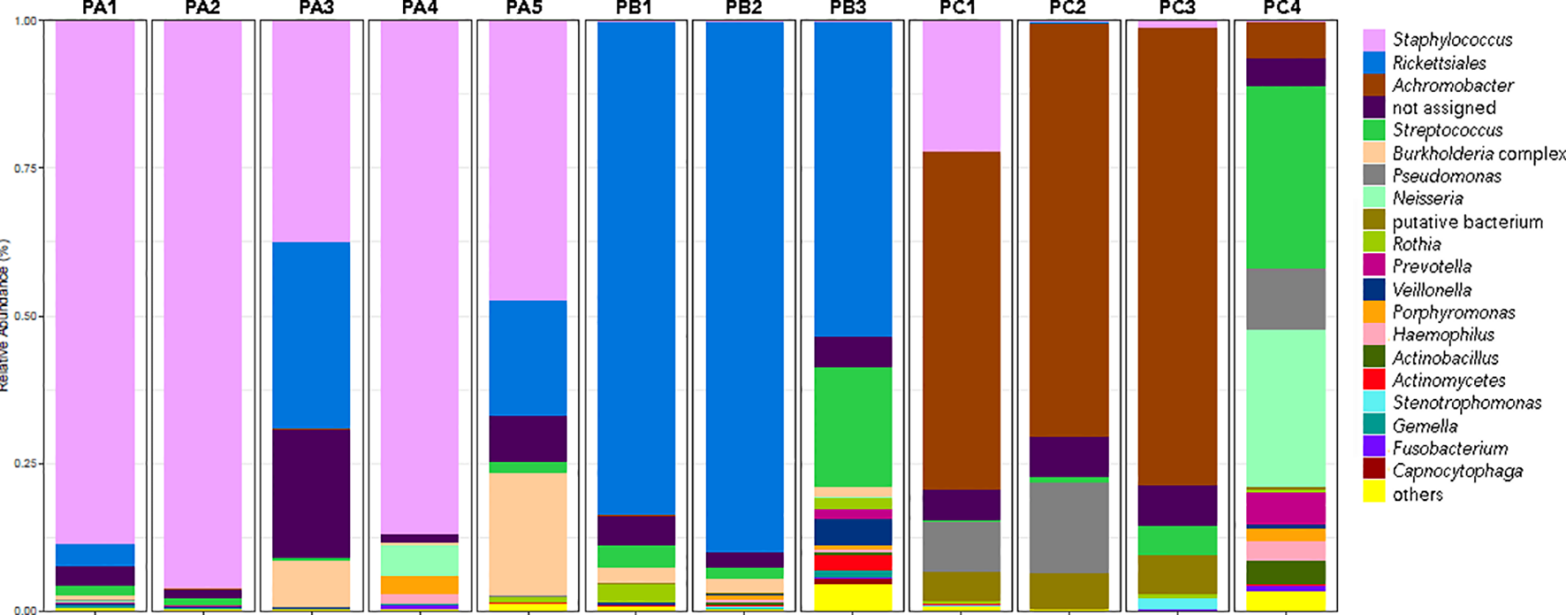
Shifts in CF patient-associated bacteria expressed by the top 21 most prevalent OTUs.

According to the metataxonomic findings, the patients presented variable bacterial compositions (**Figure 1**). For patient A, the high prevalence of *Staphylococcus* was notorious among all sputum samples evaluated. Lower amounts are shown for the *Rickettsiales* and *Burkholderia* groups in samples PA1, PA3, and PA5.

For patient B, *Rickettsiales* was the main bacterial order detected, with variable levels of *Streptococcus* and *Burkholderia* genera during the study (**Figure 1**).

Patient C was chronically colonized by *Achromobacter*, but *Staphylococcus* sp. was also detected in PC1 and PC3 samples, with a higher prevalence in the former. *Pseudomonas* genus was present in all but PC3 sputum samples. The PC4 sample was the most diverse, with the additional detection of *Streptococcus, Neisseria, Prevotella, Actinobacillus*, and *Haemophilus* (**Figure 1**).

**Figure 2:**
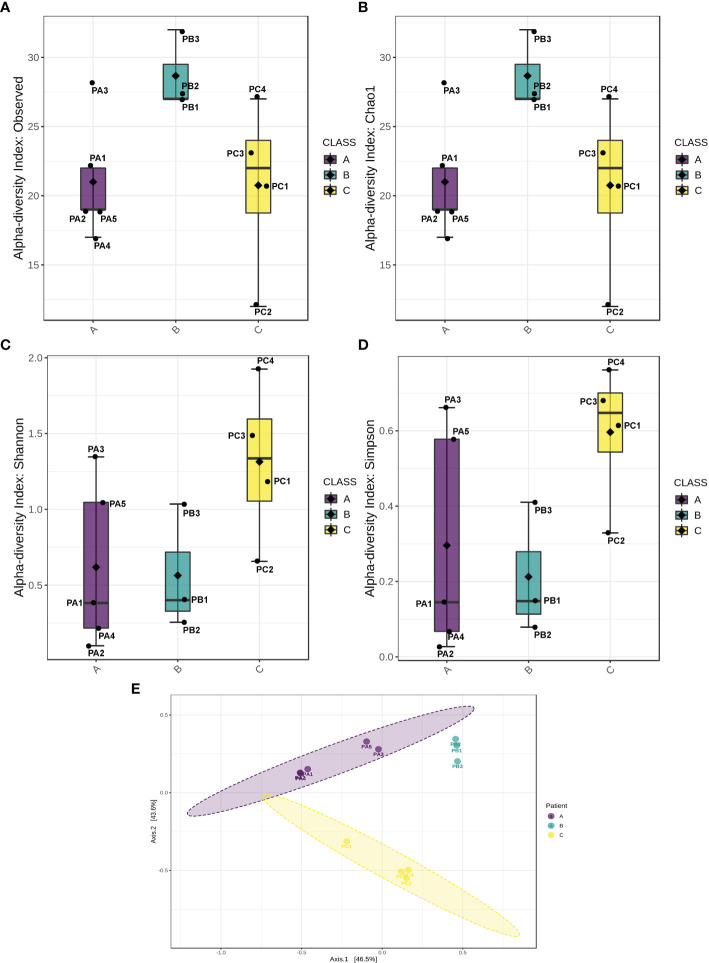
Biological diversity indexes. Indexes of *α*-diversity (intrasample comparison) represented by observed OTUs **(A)**, Chao1 **(B)**, Shannon **(C)**, and Simpson indexes **(D)**. **(E)** Index of *β*-diversity (intersample comparison).

Regarding intraspecies diversity (*α*-index), it was higher for patient B samples, as revealed by the larger number of different OTUs detected (**Figure 2A**). The theoretically expected diversity expressed by the Chao1 index (Kim et al., 2017)⁠ was very close to the OTU index observed in this study (**Figure 2B**), indicating the good quality of sampling, which means that the number of groups undetected was similar to the number of groups estimated to be undetected at random. However, the lower values of Shannon and Simpson indexes (**Figures 2C, D** **)** measured for patient B and patient C samples indicate that the majority of OTUs harbored by these patients were represented by rare groups, which is corroborated by the observation of the low relative abundances of the minor OTUs (**Figure 1**). Overall, the higher values observed for Shannon and Simpson metrics for patient C revealed a well-established microbiome, with a higher diversity in comparison with other patients, indicating a uniform relative abundance of OTUs. The comparison among the microbiota of patients (*β*-diversity) rendered a pattern of clustering per patient, indicating that each individual presented a distinguishable bacterial profile (**Figure 2E**).

**Figure 3:**
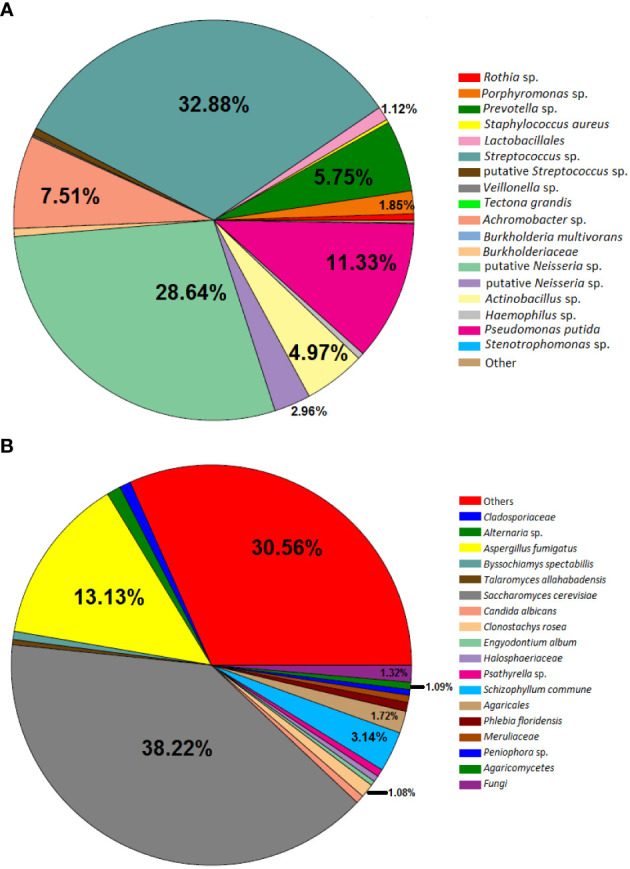
Pie chart depicting bacteria **(A)** and fungi **(B)** detected in Patient C in the sample PC4. The slices without the relative abundance information represent the taxa that have less than 1% relative abundance.

Concerning the results of metataxonomics based on ITS gene markers, fungi were detected only in the PC4 sputum sample. This patient exhibited most reads assigned to *Saccharomyces cerevisiae*, *Aspergillus fumigatus*, and *Schizophyllum* sp. (**Figure 3**). Notably, the fungi were present in the sample with the highest bacterial diversity (**Figures 1**, **2C, D**).

**Figure 4:**
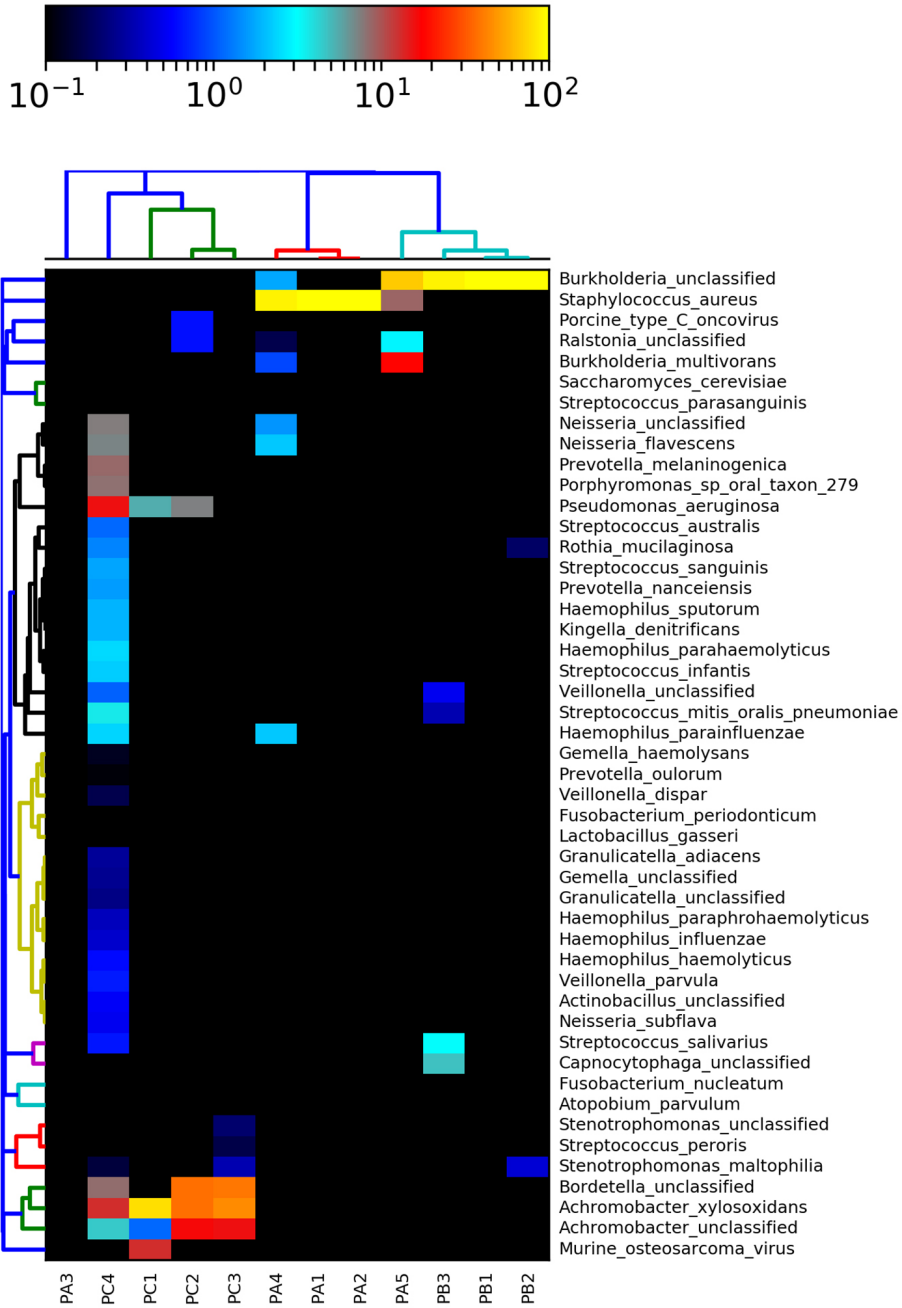
Species-level taxonomic assignment of fungi, bacteria, and viruses in the microbiome based on metagenomics data. Some taxa were not assigned to the species level, so only the genus level was expressed.

Clustering analysis based on metagenomic shotgun data revealed the occurrence of a particular microbiota in each patient, and clinical specimens presented complex microbial compositions (**Figure 4**). Almost all samples of patient A were clustered together, except for the PA3 sample, possibly due to the “not assigned” OTUs. Additionally, the PA5 sample presented high levels of *Burkholderia* complex OTUs, analogous to some samples of patient B, justifying its proximity with the patient B cluster (**Figure 4**). As patient C exhibited a very particular microbial profile, the samples were clustered independently from the other subjects.

Exploring deeply the shotgun data, the patient A microbiota was characterized by the presence of *S. aureus* during the entire study and, in lower proportions, the cooccurrence of *Neisseria* sp., *Neisseria flavescens*, *Haemophilus parainfluenzae*, *Ralstonia* sp., and *Burkholderia* sp. (**Figure 4**).

The patient B microbiota was marked by the dominance of *Burkholderia cepacia* complex and the co-occurrence of *Rothia mucilaginosa*, *Stenotrophomonas maltophila*, *Veillonella* sp., *Streptococcus mitis/oralis/pneumoniae*, *Streptococcus salivarius*, and *Capnocytophaga* sp. (**Figure 4**).

Finally, patient C exhibited the most diverse microbiota, as illustrated by **Figures 2C, D**. *Achromobacter* sp. and *A. xylosoxidans* were present in all sputum samples. *Pseudomonas aeruginosa* and *Bordetella* sp. were observed in 75% (n = 3) of the sputum samples. PC4 was the most diverse sample, with the cooccurrence of low relative ratios of species from the genera *Haemophilus*, *Streptococcus*, *Kingella*, *Prevotella*, *Neisseria*, *Porphyromonas*, *Veillonella*, *Actinobacillus*, and *Rothia* (**Figure 4**).

In summary, the shotgun clustering snapshot corroborated the metataxonomic assignment of OTUs and the *β*-diversity measurements, which unequivocally translated the real community structure assemblage and justified the high microbial diversity found in patient C.

### Virulence and Antibiotic Resistance Genes

**Figure 5:**
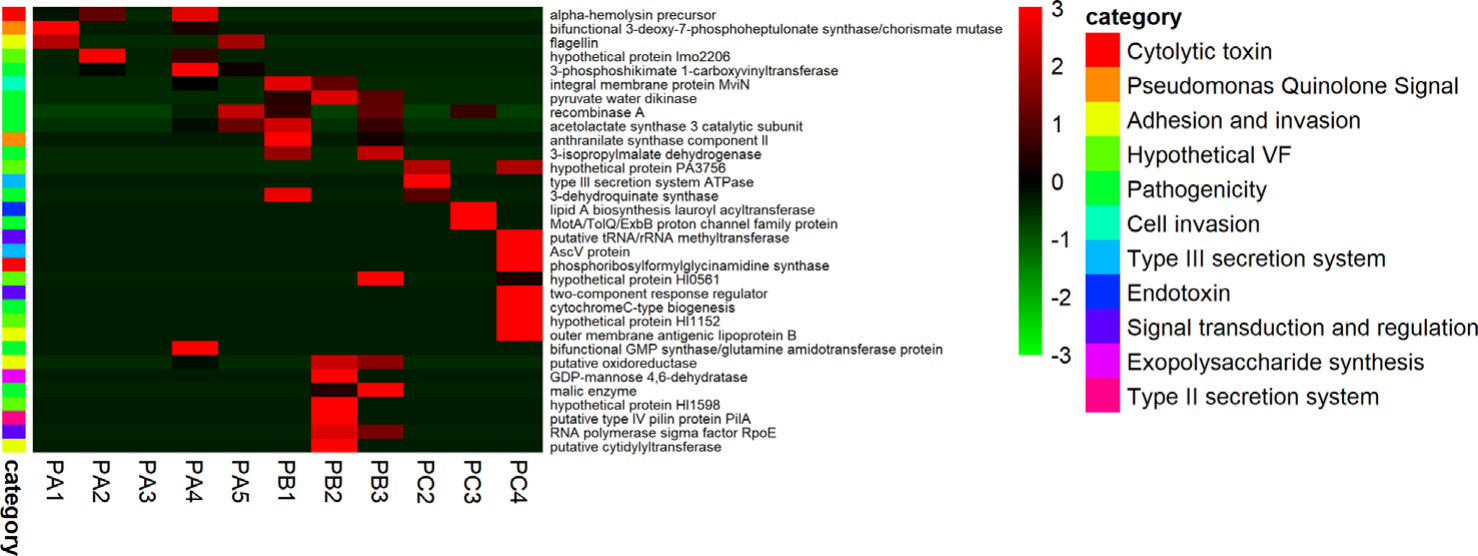
Disease-related potential. Virulence sequences of fragmented genes detected on the CF-associated microbiome.

The alignment of quality-processed reads against the custom marker genes database created from the comparison of UniRef90 and Victor databases detected sets of reads matching against 33 coding genes related to putative virulence factors. As observed for microbial succession, the prevalence of specific virulence determinants was highly variable according to each patient sampling time. In general, patient A exhibited high amounts of reads related to *Pseudomonas* quinolone signal (PQS), adhesion/invasion, cytolytic toxins, and general pathogenicity-enhancing effectors. In contrast, the most diverse microbiota in terms of virulence repertoire were those from patients B and C, which varied in terms of the presence of type II secretory systems and exopolysaccharide synthesis, presence of type III secretory systems, endotoxin synthesis and cytolytic potential (**Figure 5**).

**Figure 6:**
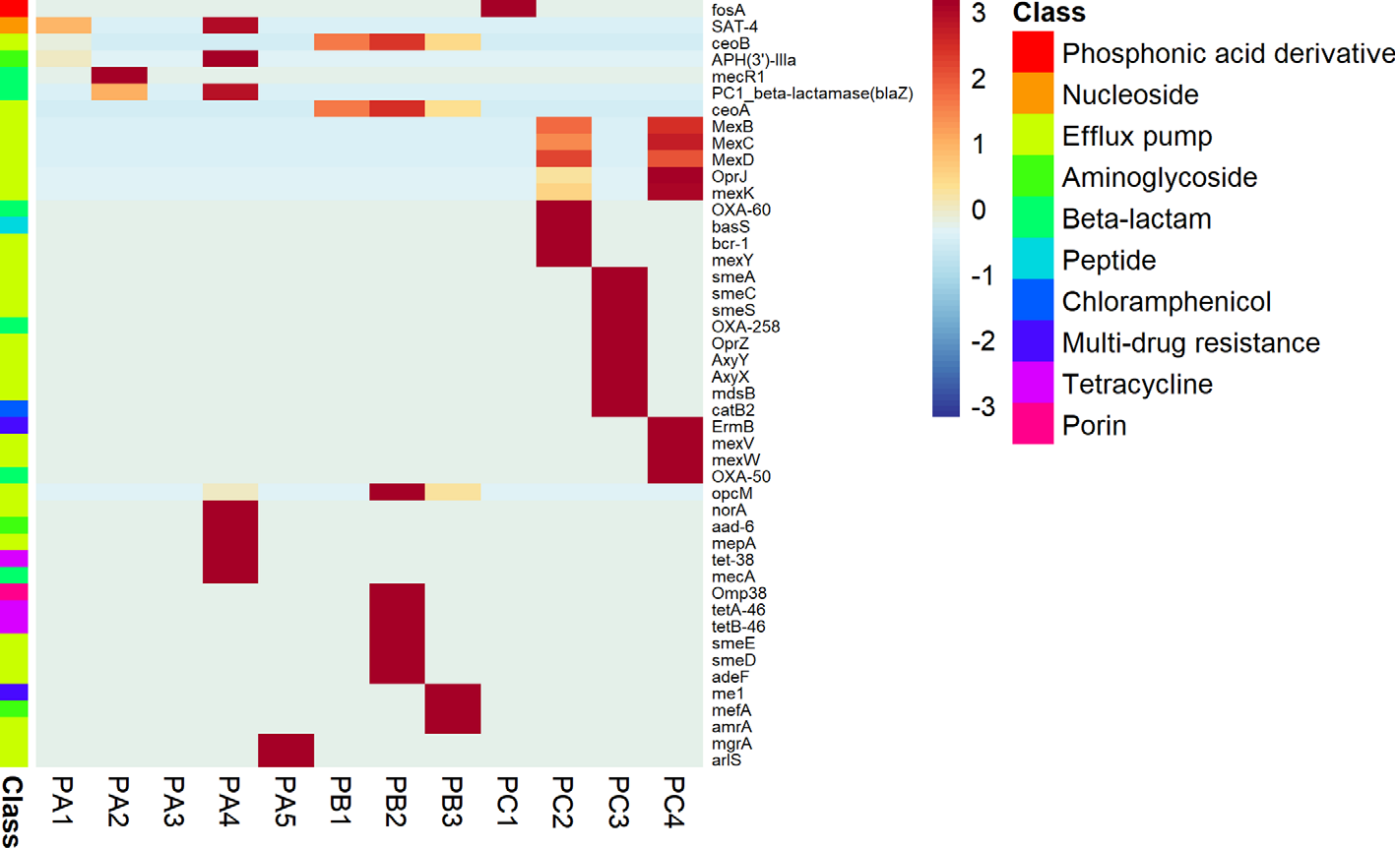
Disease-related potential. ARG sequences of fragmented genes detected on the CF-associated microbiome.

Considering the ARG profiles, the alignments against the custom CARD and UniRef90 databases returned sets of reads matching 46 putative ARG coding genes. The antimicrobial profiles were variable according to the patient, but each one presented a huge diversity of efflux pump determinants. Patient A presented reads matching to nucleoside, beta-lactam, and aminoglycoside antibiotic classes, while the patient B microbiome was more related to general multidrug resistance effectors, porins with reduced permeability to antibiotics and tetracycline resistance. Finally, patient C presented genes conferring resistance to phosphonic acid derivatives, aminoglycosides, beta-lactams, chloramphenicol, and multidrug-related drugs (**Figure 6**).

## Discussion

The lung microbiomes of three young patients with CF with compromised pulmonary function were studied. The investigation is particularly interesting once the microbial community is more dynamic in infants than in adults (Cox et al., 2010; Klepac-Ceraj et al., 2010; Frayman et al., 2017). Virulence factors and antibiotic resistance genes were also explored.

The bacteria isolated from sputum samples were *S. aureus*, *P. aeruginosa*, and *B. cepacia* complex from patient A; *B. cepacia* complex from patient B; and *Achromobacter* sp., *A. xylosoxidans*, *Stenotrophomonas maltophilia*, *P. aeruginosa*, and *Chryseobacterium indologenes* from patient C. Although metataxonomic and metagenomic analyses identified most of these species, reads of *P. aeruginosa* were not observed in patient A sputum samples. Additionally, metataxonomic and metagenomic analyses identified species of the genera *Streptococcus*, *Neisseria*, *Rothia*, *Prevotella*, and *Haemophilus*, among others. The differences between identification by both culture isolation and HTS strategy show that the culture complexity and fastidious nutritional requirements of some pathogens can lead to non-isolation in standard microbial culture (Zemanick et al., 2017), and in the intrinsic composition of metagenomic samples with a high proportion of host-DNA, which sometimes encompasses more than 99% of the dataset, as was observed in this study, limiting the resolution of community composition. A recent study advocates the use of culture-enrichment metagenomics associated with the traditional HTS methods to improve the identification of low-abundant organisms and microbiome characterization, which may overcome this limitation (Lim et al., 2014).

As mentioned above, patient B showed an early poor outcome that culminated in patient death. The same patient had the lowest lung microbiota diversity, and studies have linked the decreased lung microbiota diversity with a reduction in lung function (Cuthbertson et al., 2020), which can justify the poor outcome. Additionally, the increase in microbiota dominance occurs in the loss of microbiota diversity (Cuthbertson et al., 2020). Members of *Pseudomonas*, *Staphylococcus*, and *Achromobacter* genera found in this study are potential pathogenic microorganisms and are frequently isolated from the airways of CF patients (Coburn et al., 2015; Losada et al., 2016)⁠. *B. cepacia* complex members were also found in this study. These members are most frequently isolated from patients with CF and generally linked to pulmonary deterioration (Coburn et al., 2015), high risk to the host due to multidrug resistance, easy transmissibility, the potential to cause septicemia, and an unpredictable clinical outcome, varying from an asymptomatic infection up to fatal pneumonia known as “cepacia syndrome” (Zlosnik et al., 2015; Hector et al., 2016)⁠. A previous study in the same hospital showed patients with good clinical outcomes and favorable options for antibiotic therapy (da Costa Capizzani et al., 2017). Even so, possibly the highly impaired lung functions of these young patients are related to the early presence of very pathogenic bacteria, related to adverse outcomes.

Patient A’s lung microbiome had a prevalence of *S. aureus* and *B. cepacia* complex, which was shown by culture isolation, metataxonomics, and metagenomics from the sputum samples*. S. aureus* is a common pathogen recovered from CF samples, and the association of this species with exacerbations in CF is evidenced by high levels of inflammation due to stimulus in interleukin-6 (IL-6) recruitment and eventually provoking lung damage (Hurley, 2018).

The patient B microbiome was primarily dominated by *B. cepacia* complex. This species was identified by both culture isolation and metataxonomics/metagenomics. It is commonly identified in CF patients and represents a high risk to the host due to the carriage of several virulence factors (VFs), such as the expression of secretion systems, several colony variants, and the presence of lipopolysaccharides, which are VFs frequently related to pandrug-resistance phenotypes (Lopes et al., 2015).

*Achromobacter* *xylosoxidans* is also a prevalent CF-related microbe and has been considered an emergent bacterium. It was highly detected in patient C lung microbiota, although *A. insuavis* and *A. ruhlandii* species are currently known as key pathogens in CF disease as well. These bacteria present resistance against the innate immune response, high plasticity in front of antimicrobial agents, may acquire resistance in short periods of exposure to antimicrobials, and can alter the expression of a set of genes to promote chronic infection (Edwards et al., 2017).

According to the literature review, the lung microbiota in CF disease may be divided into emergent pathogen microbiota and core microbiota. Commonly, the core microbiota of CF pediatric patients is formed by the genera *Streptococcus*, *Rothia, Prevotella*, *Actinomyces*, *Veillonella*, *Gemella*, *Neisseria*, and *Haemophilus*, which are commensal bacteria and not necessarily related to CF disease (Prevaes et al., 2017). Metataxonomic and metagenomic analyses identified the canonical core CF microbiome species from the genera *Streptococcus, Neisseria, Rothia, Prevotella*, and *Haemophilus*, among others, in our study. Association studies showed that the taxa *Pseudomonas aeruginosa, Rothia mucilaginosa*, and *Streptococcus pneumoniae* were related to more deteriorated lung function (Paganin et al., 2015). *Rothia* sp. and *Streptococcus* sp. were detected at variable levels by metataxonomics in all sputum samples, but *Pseudomonas aeruginosa* was detected only in the patient C microbiome, concomitant with high levels of *Achromobacter* spp. The presence of all these taxa in the CF microbiome could predict a progressive loss in lung function.

Regarding the core microbiota, attention must be paid to *H. influenza*, which is a bacterium that naturally colonizes the respiratory tract during infancy but has recently been reported as an emergent pathogen frequently related to acute respiratory infections, contributing to the local inflammatory response and premature lung damage. The main concern with *H. influenza* pathogenicity is its strong ability for biofilm formation, which leads to high tolerance to antibiotics (Cardines et al., 2012)⁠. On the other hand, there are also reports describing the protective effect of *Haemophilus* spp. colonization to the lungs of adolescent patients with CF (Hector et al., 2016)⁠. Additionally, *S. maltophilia* and *Ralstonia* sp. were emergent pathogens detected among the sputum samples (considering both approaches of OTU assignment). Recent publications suggest that *S. maltophilia* plays several roles in lung damage, which can culminate with disease exacerbation, lung transplantation, and even death (Waters et al., 2013)⁠. The highly successful adaptation of *S. maltophilia* to CF is related to intrinsic antibiotic resistance, a decrease in biofilm formation, and pathogenicity in chronic disease. Additionally, *S. maltophilia* exhibits marked phenotypic and genotypic heterogeneity and complex regulatory networks among *S. maltophilia* clones in CF disease, which makes it difficult to identify therapeutic strategies to eradicate this bacterium (Esposito et al., 2017)⁠.

*Ralstonia* spp. infections in CF are still poorly reported, probably because it is underestimated, since this pathogen can grow on *Burkholderia cepacia* selective agar, generally with high levels of contamination by other pathogens, such as *P.* *aeruginosa* and *S. aureus* (Green et al., 2017). The genus *Ralstonia* is a common cause of nosocomial infections in CF patients due to contamination of hospital devices (*i.e.* respiratory therapy) and distilled water used to prepare solutions to be injected in patients. As infection is commonly related to polymicrobial communities in CF, it is difficult to establish the role of *Ralstonia* species in pathogenicity, but a high prevalence of this potential pathogen has been reported in the CF microbiome when the lung microbiome diversity is too low (Prior et al., 2017).

Thus, despite the low number of samples here, the airway of the CF patients presented dominant bacterial genera and inter-individual variability in microbial community composition and diversity, confirming the results of individual signatures of multiple species recorded in other studies (Delhaes et al., 2012; Losada et al., 2016; Güemes et al., 2019). The individual variations of microbial composition are most likely attributed to the patient’s domestic environment and how patient’s microbiome will respond to therapeutic perturbation (Whelan et al., 2020).

The depleted mucociliary clearance and the thick mucus filling the airways of CF patients easily trap the inhaled fungal conidia. *A. fumigatus* is the most prevalent species involved in CF colonization and disease (Warris et al., 2019) and was culture isolated at a high frequency from the sputum samples of patients A and C. Additionally, *A. fumigatus* with the same sequence type was observed in different sputum samples of each patient. Persistent colonization occurs when the same species is identified twice or more times in the same year (Saunders et al., 2016). Thus, the lung airways of patients A and C have persistent colonization of the lung airways by *A. fumigatus*. On the other hand, transient colonization of *Penicillium* sp., *Hanseniaspora* sp., *Torulaspora delbrueckii*, and *Talaromyces amestolkiae* was observed in the lung airways of patient B once a single culture isolation was registered (Saunders et al., 2016). The isolation of *A. fumigatus* in respiratory secretions of CF patients is a common occurrence. However, the mean age of the patient at the date of the first isolation of *A. fumigatus* ranges from 9 to 16 years old (Whelan et al., 2020). Our results reinforce the literature data once 9-year-old patient B has no *A. fumigatus* isolation from sputum samples, whereas older patients (A and C) present persistent colonization by this fungal species.

The culture method was able to recover fungal species in most sputum samples once it is an enrichment-based strategy and appropriate selective media with longer incubation times, which are not routinely performed in clinical microbiology labs (Hong et al., 2017; Martín-Gómez, 2020)⁠. On the other hand, metataxonomic and metagenomic analyses detected fungal reads in only one sputum sample (PC4). It is important to note that fungi are generally present in low amounts in the lung microbiome of CF patients, representing approximately 1% of the total OTUs (Losada et al., 2016)⁠, hampering the identification of these microorganisms by the culture-independent method. Thus, considering only metagenomics data, without the enrichment of gene markers by metataxonomics, few fungal reads were assessed, suggesting that metagenomics may underestimate fungi detection since the higher bacterial biomass and accelerated proliferation may overwhelm the fungi reads. The concordance between the molecular methods was in the detection of *S. cerevisiae* reads. *S. cerevisiae* was previously identified in sputum from CF patients (Güngör et al., 2013; Ziesing et al., 2016). A recent study evaluated the fungal composition in low- and high-severity CF patients against healthy subjects through isolation methods and found that *S. cerevisiae* was present in high-severity or healthy subjects, but its meaning in the context of CF physiopathology is unknown.

It is important to highlight the high diversity of bacterial species in the PC4 sample in comparison with the other samples. However, the copresence of fungal members and its influence on bacterial diversity is a matter that needs future investigation. The high microbiome diversity is related to low ratios of pathogenic bacteria and to an improvement in lung function (Carmody et al., 2015; Losada et al., 2016). However, the presence of pathogenic fungi, such as *A. fumigatus*, one of the major fungi identified in PC4, may not be positive since there is evidence of its role in CF pathophysiology causing a significant decrease in FEV-1 and being frequently correlated with inflammatory episodes (Eickmeier et al., 2020).

*Schizophyllum commune* was the third most prevalent fungal OTU detected in the patient C metagenome. This basidiomycete is an environmental fungus that commonly grows on organic matter. However, a novel report linked this microorganism to allergic bronchopulmonary mycosis (ABPM) and pulmonary fungal ball (Chowdhary et al., 2013), characterizing it as an emergent pathogen.

Regarding the other fungal taxa detected, to the best of our knowledge, there are no conclusive reports on their influence in CF disease. The well-known yeast *C. albicans* is frequently isolated from the sputum culture of CF patients. It is believed that the use of inhaled steroids and antibiotics predisposes *Candida* sp. colonization (Williams et al., 2016).

In this work, we observed a wide diversity of VFs and ARGs found in all CF microbiomes. As pointed out by a previous assay, the abundance of VFs and ARGs in CF microbiomes are not directly related to a specific bacterial group but are spread among several members of the CF community, indicating that the emergence of these traits should be linked to a particular clinical condition of the CF patient (normal, mild or severe) and enhanced by the antibiotic therapy treatment provided in care units for long-term periods (Bacci et al., 2017).

Antibiotic stewardship indeed may potentiate resistance by triggering downstream responses on the bacterial community, such as the upregulation of efflux pumps in biofilm formation, making bacteria immune to antimicrobials or enhancing the rates of horizontal gene transfer among bacteria (Sherrard et al., 2014). On the other hand, susceptible bacteria can acquire temporary resistance to antibiotics due to microbial interactions in the lung environment. Resistant bacteria can modify antibiotics, diminishing their efficacy, or biofilm formation may limit and dilute the minimum inhibitory concentration values resulting in sublethal doses or fungi that may protect bacteria from forming hyphal barriers (Vandeplassche et al., 2019).

A recent study suggests that a therapy focusing on eliminating anaerobic bacteria re-establishes the canonical CF microbiota, improving lung function and respiratory health (Silveira et al., 2020). At the same time, evidence of a therapy targeted to the weakest cross-feeder, a susceptible bacterium that feeds the entire microbial community, reduces the viability of the resistant ones, improving treatment efficacy (Adamowicz et al., 2018). Currently, the simple detection of virulence factors and antibiotic resistance genes in CF microbiomes is unmeaning if microbial interactions and community response to therapy are not considered.

In summary, the lung airway microbiome of three young CF patients with fungal colonization presents a personal signature with low variation in the microbiome across pulmonary exacerbations and a core set of virulence factors and antibiotic resistance genes. Understanding the microbial community is crucial to improve therapy because it may have the opposite effect, restructuring the pathogenic microbiota. Future studies focusing on the influence of fungi on bacterial diversity and microbial interactions in CF microbiomes will be welcome to fulfill this huge gap in fungi influence in CF physiopathology.

## Data Availability Statement

The dataset generated for this study can be found in GenBank accession MT886813 to MT886844, MT891039 to MT891061, and MT911441 to MT911463. The metataxonomic sequence data are available in Bioproject PRJNA644204. The shotgun data are available in Bioproject PRJNA644285.

## Ethics Statement

The studies involving human participants were reviewed and approved by the Ethics Committee of “Faculdade de Ciências Farmacêuticas de Ribeirão Preto da Universidade de São Paulo” (FCFRP-USP), under protocol number 2.492.043, in accordance with the HC-FMRP-USP as a co-participating institution. Written informed consent to participate in this study was provided by the participants’ legal guardian/next of kin. Written informed consent was obtained from the minor(s)’ legal guardian/next of kin for the publication of any potentially identifiable images or data included in this article.

## Author Contributions

OA performed the data curation, formal analysis, investigation, methodology, visualization, and wrote, reviewed, and edited the original draft. CC wrote, reviewed, and edited the manuscript. LuT was in charge of the methodology. PB was in charge of the methodology and wrote, reviewed, and edited the manuscript. AC conceptualized the study and wrote, reviewed, and edited the manuscript. EM supervised the study and wrote, reviewed, and edited the manuscript. LiT was responsible for the formal analysis, visualization, and writing and reviewing the manuscript. MK conceptualized the study, and was in charge of the methodology, project administration, supervision, formal analysis, funding acquisition, project administration, resources, visualization, writing, reviewing, and editing original draft. All authors contributed to the article and approved the submitted version.

## Funding

This work was funded by The São Paulo Research Foundation—FAPESP (Grant # 17/25300-8). OA is grateful to FAPESP for the Ph.D. fellowship (Grant #17/13759-6). This work was also partially funded by Coordenação de Aperfeiçoamento do Ensino Superior—CAPES (Finance code-001) and Conselho Nacional de Desenvolvimento Científico e Tecnológico—CNPq.

## Conflict of Interest

The authors declare that the research was conducted in the absence of any commercial or financial relationships that could be construed as a potential conflict of interest.
